# Developmental and Degenerative Features in a Complicated Spastic Paraplegia

**DOI:** 10.1002/ana.21923

**Published:** 2009-11-30

**Authors:** M Chiara Manzini, Anna Rajab, Thomas M Maynard, Ganeshwaran H Mochida, Wen-Hann Tan, Ramzi Nasir, R Sean Hill, Danielle Gleason, Muna Al Saffar, Jennifer N Partlow, Brenda J Barry, Mike Vernon, Anthony-Samuel LaMantia, Christopher A Walsh

**Affiliations:** 1 Department of Neurology and Howard Hughes Medical Institute, Beth Israel Deaconess Medical Center, Harvard Medical SchoolBoston, MA; 2 Genetic Unit, Directorate General of Health Affairs, Ministry of HealthMuscat, Sultanate of Oman; 3 Department of Cell and Molecular Physiology and UNC Neuroscience Center, University of North Carolina School of MedicineChapel Hill, NC; 4 Pediatric Neurology Unit, Massachusetts General HospitalBoston, MA; 5 Division of Genetics and Manton Center for Orphan Disease Research, Children's Hospital BostonBoston, MA; 6 Division of Developmental Medicine, Children's Hospital BostonBoston, MA

## Abstract

**Objective:**

We sought to explore the genetic and molecular causes of Troyer syndrome, one of several complicated hereditary spastic paraplegias (HSPs). Troyer syndrome had been thought to be restricted to the Amish; however, we identified 2 Omani families with HSP, short stature, dysarthria and developmental delay—core features of Troyer syndrome—and a novel mutation in the *SPG20* gene, which is also mutated in the Amish. In addition, we analyzed *SPG20* expression throughout development to infer how disruption of this gene might generate the constellation of developmental and degenerative Troyer syndrome phenotypes.

**Methods:**

Clinical characterization of 2 non-Amish families with Troyer syndrome was followed by linkage and sequencing analysis. Quantitative polymerase chain reaction and in situ hybridization analysis of *SPG20* expression were carried out in embryonic and adult human and mouse tissue.

**Results:**

Two Omani families carrying a novel *SPG20* mutation displayed clinical features remarkably similar to the Amish patients with Troyer syndrome. *SPG20* mRNA is expressed broadly but at low relative levels in the adult brain; however, it is robustly and specifically expressed in the limbs, face, and brain during early morphogenesis.

**Interpretation:**

Null mutations in *SPG20* cause Troyer syndrome, a specific clinical entity with developmental and degenerative features. Maximal expression of *SPG20* in the limb buds and forebrain during embryogenesis may explain the developmental origin of the skeletal and cognitive defects observed in this disorder. ANN NEUROL 2010;67:516–525

Hereditary spastic paraplegias (HSPs) comprise several disorders commonly divided into 2 subgroups: “pure” HSPs characterized by progressive spasticity in the lower limbs due to pyramidal tract degeneration and “complicated” HSPs, where lower limb spasticity is associated with a variety of other neurological signs and clinical features. Complicated HSPs are clinically heterogeneous, mainly autosomal recessive syndromes, frequently described and mapped in sporadic families within inbred populations.^1^–^3^ Because of this heterogeneity, diagnosis and recommendations for genetic testing in these disorders have been daunting tasks.

Troyer syndrome (Online Mendelian Inheritance in Man #275900) is a complicated HSP associated with short stature, skeletal abnormalities, dysarthria, and developmental delay, first described in the Old Order Amish.^4^, ^5^ Since the original description in 1967, several Troyer-like syndromes have been reported,^6^–^9^ but they often differed from classical Troyer syndrome in their neurological or skeletal features. The Amish founder mutation is a single nucleotide deletion in the *SPG20* gene,^10^ leading to the loss of the spartin protein^11^; however, no additional *SPG20* mutations were subsequently identified,^5^ and it was suspected that Troyer syndrome may be restricted to the Amish.

We identified an Omani kindred presenting with clinical features resembling Troyer syndrome. All affected Omani individuals had a novel homozygous null mutation in *SPG20*, and their clinical descriptions matched closely to those of Amish Troyer syndrome individuals of comparable ages. Because Troyer syndrome is associated with developmental features, such as short stature, skeletal abnormalities, and global developmental delay, we investigated the sites of action of *SPG20* during development in humans and mice. *SPG20/Spg20* expression in the adult is relatively modest and widespread in the nervous system; however, maximal and focal expression is observed in the embryonic limb buds, face, and forebrain during early morphogenesis, which might explain the developmental phenotypic changes involving the extremities, face, and brain.

## Subjects and Methods

### Enrollment and Clinical Studies

The subjects belonged to 2 families residing in a remote region of Oman and were originally ascertained by a local clinical geneticist. Detailed family and medical histories were obtained by a genetic counselor, who is a native Arabic speaker, and a developmental pediatrician, also a native Arabic speaker, conducted a developmental assessment. A pediatric neurologist performed a standard neurological examination, and a second clinical geneticist obtained anthropometric measurements. The height of all subjects and head circumference of subjects under the age of 36 months were plotted on the Centers for Disease Control and Prevention 2000 growth charts,^12^ and head circumference of subjects above 36 months and all other anthropometric measurements were plotted on standard charts.^13^ Written, informed consent was obtained from the subjects or their legal guardians. The Ministry of Health in Oman and the institutional review board of Children's Hospital Boston approved this study.

### Linkage Analysis

Genomic DNA was purified from lymphocytes separated from peripheral blood using commercial kits (Qiagen, Valencia, CA). Six hundred nanograms of genomic DNA was used to hybridize Affymetrix Human SNP Array 6.0 at the Genomic Analysis Core of the University of North Carolina (UNC) Neuroscience Center of the UNC School of Medicine, Chapel Hill, North Carolina. After removal of low-quality calls and of Mendelian and non-Mendelian errors using Merlin software,^14^ the single nucleotide polymorphism (SNP) data were analyzed using Allegro software^15^ under a fully penetrant autosomal recessive model. For microsatellite analysis, highly polymorphic microsatellite markers were chosen from the Marshfield database in the University of California at Santa Cruz Genome Browser. Fluorescently labeled polymerase chain reaction (PCR) primers (Applied Biosystems, Foster City, CA) were used to amplify DNA samples using standard conditions, and PCR products were resolved on an Applied Biosystems 3130xl Genetic analyzer.

### SPG20 Sequencing Analysis

Sequencing of *SPG20* (NM_015087; NM_001142294-6) coding region was performed by SeqWright (Houston, TX) on PCR products after amplification of genomic DNA. PCR primers were designed for each exon including at least 50bp of flanking intronic sequences. Primer sequences are available on request. Once a putative change was identified, at least 96 control DNAs (192 alleles) were tested to exclude the possibility of a benign polymorphic change.

### Patient Cell Lines and Mutated Protein/mRNA Detection

Transformed lymphoblastoid cell lines were established from peripheral blood of affected and unaffected individuals at the Partners Center for Personalized Genetic Medicine (Cambridge, MA). Cell lines were maintained in RPMI medium supplemented with 10% fetal bovine serum, glutamine (2mM) and penicillin/streptomycin (100U/100μg) (all from Gibco, Grand Island, NY). Protein lysates were obtained by boiling a cell pellet in Laemmli sample buffer (Bio-Rad Laboratories, Hercules, CA). Western blotting was performed using standard protocols on Bio-Rad equipment. Presence of spartin was assessed using a rabbit anti-SPG20 antibody (Proteintech Group, Chicago, IL) and a goat anti-rabbit IRDye-800CW secondary antibody (LI-COR Biosciences, Lincoln, NE) detected on an Odyssey Imager (LI-COR Biosciences).

Total RNA was purified from patient cell lines using the mirVana RNA Isolation Kit (Ambion Applied Biosystems, Foster City, CA). Random primed cDNA was generated using reverse transcriptase (Promega, Madison, WI) and analyzed by quantitative PCR (qPCR) using SYBR Green reagents (Applied Biosystems) on an ABI 7500 qPCR platform as previously described.^16^ Primer sequences are available in Supplementary Table.

### SPG20/Spg20 Expression Analysis

cDNA samples from human fetal and adult postmortem brain tissue were obtained commercially (BioChain Institute, Hayward, CA and Clontech, Mountain View, CA). cDNA from embryonic and postnatal mouse brains was generated as described above. qPCR was performed as described above (see Supplementary Table for primer sequences). DNA templates for in situ hybridization probes were cloned using a 550bp fragment of the *Spg20* coding sequence using the following primers: Spg20-BamHI 5′-ctacatggatccgatatcaaccggaggagcagccaaagtcagc-3′ and Spg20-SalI 5′-gtccttgtcgactgcccctggcttctcttcctccacctg-3′. Digoxigenin-labeled riboprobes were synthesized and in situ hybridization was performed as previously described.^17^ Video images of the hybridized sections were obtained on a Wild Leitz (Heerbrugg, Switzerland) photomicroscope or Leica (Nussloch, Germany) DMR microscope under consistent illumination conditions.

## Results

### Identification of a Novel SPG20 Mutation in an Omani Kindred

**FIGURE 1:**
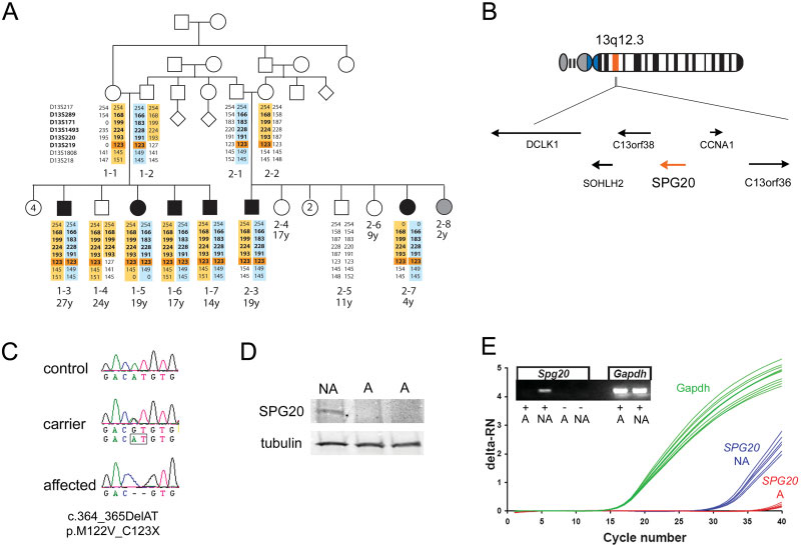
Affected individuals carry a homozygous null mutation in the *SPG20* gene. (A) Pedigree of 2 related Omani families affected with short stature, spasticity, dysarthria, and developmental delay. Affected individuals are in black and unaffected in white. The status of individual 2-8 (in gray) could not be determined, because she is too young to properly assess neurological symptoms. All numbered individuals were examined, but genomic DNA was only collected from individuals for whom microsatellite analysis is shown. Microsatellite analysis revealed common maternal (in yellow) and paternal (in blue) haplotypes in all affected. Microsatellite markers in the linkage region identified by the single nucleotide polymorphism analysis are in bold. One homozygous marker (D13S219) is common to all affected individuals (highlighted in orange). (B) The homozygous region contains 6 genes, including *SPG20* (in orange). (C) The affected individuals carried a homozygous 2bp deletion in *SPG20*, which was present in heterozygosity in the carriers. (D) Western blot analysis of patient cell lines showed that full-length SPG20 protein is missing in the affected individuals (A) compared with a nonaffected noncarrier sibling (NA). (E) Quantitative polymerase chain reaction analysis of cDNA from the patient cell lines indicated that *SPG20* mRNA is not present in the affected individual. deltaRN = signal magnitude expressed by the difference in the normalized reporter (RN) values.

We examined 2 related Omani families presenting with short stature, dysarthria, motor and cognitive developmental delay, and increased muscle tone (families 1 and 2 in Fig 1A). The fathers (individuals 1-2 and 2-1) were brothers, and the mothers (1-1 and 2-2) were aunt and niece. Family 1 had 9 children: 4 were reportedly unaffected females, who were married and lived elsewhere, 1 was a male (1-4), who was unaffected by clinical examination, 3 were affected males (1-3, 1-6, and 1-7), and 1 was an affected female (1-5). Family 2 had 8 children: 4 unaffected girls and 1 unaffected boy, 3 of whom were present at the time of our evaluation (2-4, 2-5 and 2-6), 1 affected male (2-3), 1 affected female (2-7). The youngest child (2-8) was small for her age and could neither talk nor walk, but did not display overt neurological symptoms; we were not able to obtain DNA from her for genotyping or sequencing.

Genomic DNA was obtained from most individuals in the pedigree and hybridized to Affymetrix Human SNP Array 6.0 chips for genome-wide genotyping. We suspected the presence of a homozygous ancestral mutation because the families resided in an isolated mountain village and large stretches of homozygous DNA identified in all individuals suggested some degree of consanguinity; however, we could not ascertain any shared ancestry as far as 5 generations removed from the probands by pedigree analysis. Thus, the SNP data was first analyzed following an autosomal recessive model assuming no consanguinity. Linkage analysis identified 1 region of 7.0Mb flanked by SNPs rs11619306 and rs9576104 on chromosome 13q12.3 (log of odds = 3.6). Microsatellite analysis confirmed a shared maternal and paternal haplotype in all affected individuals (see Fig 1A). Individual 1-4, who was unaffected, was homozygous for the maternal haplotype throughout most of the linkage region, excluding marker D13S219, which was homozygous on all affected individuals. At this locus, the SNP data indicated an 844kb region of homozygosity between markers rs1418987 and rs9315443, shared only by affected but not unaffected individuals. Among the 6 genes in this region (see Fig 1B), *SPG20* was the strongest candidate gene, because individuals carrying an *SPG20* mutation were affected with a remarkably similar phenotype, a complicated form of hereditary spastic paraplegia associated with short stature, dysarthria, and developmental delay, called Troyer syndrome.^4^, ^10^

Sequencing of the *SPG20* coding regions revealed a homozygous 2bp deletion (c.364_365delAT), which resulted in an amino acid substitution followed by a stop codon in the first coding exon (p.M122VfsX1; see Fig 1C). This mutation was confirmed in all affected individuals, and the inheritance pattern was confirmed in the rest of the families. This mutation was not identified in control individuals, indicating that this is not a common polymorphism.

Since the *SPG20* mutation in the Amish (c.1110delA) was reported to be a null mutation,^11^ we generated lymphoblastoid cell lines from 2 affected (1-3 and 1-5) and 1 unaffected individual (2-5). We found that the full-length spartin protein was absent in the affected individuals (see Fig 1D). We also carried out qPCR for *SPG20* on cDNA samples from 2 of the lymphoblastoid lines and could not detect any mRNA in the affected individual (see Fig 1E), indicating that this mutation is a null allele.

### Clinical Characterization of Patients Carrying a Novel SPG20 Mutation

**FIGURE 2:**
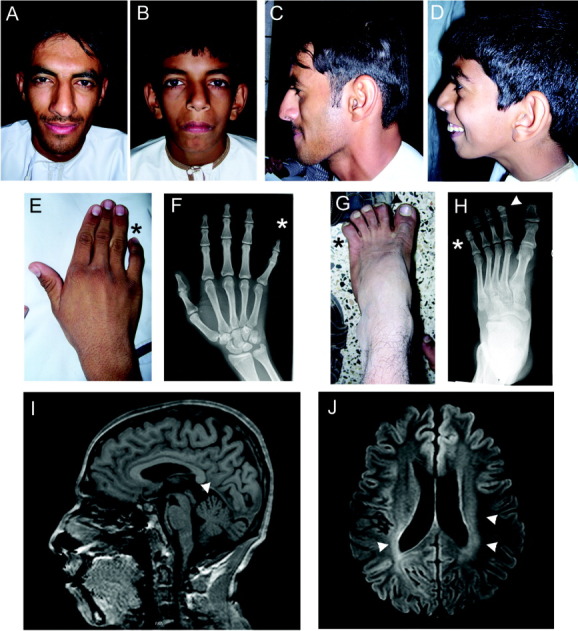
Morphological and radiological features of Omani individuals with Troyer syndrome. Comparison between (A, C) an unaffected and (B, D) an affected individual revealed (A, B) relative hypertelorism and (C, D) pronounced overbite. Examples of skeletal anomalies in the extremities: brachydactyly (short digits; E, F), camptodactyly (flexed digit; *asterisks* in E, F), hammer toes (*asterisks* in G, H), and clinodactyly (curved digit; *arrowheads* in G, H). Brain magnetic resonance imaging shows atrophy of the cerebellar vermis (*arrowhead* in I) and white matter hyperintensity in T2-weighted images, which is more prominent posteriorly (*arrowheads* in J).

To ascertain phenotypic similarities or differences among individuals carrying *SPG20* mutations, we compared the clinical presentations of the Omani individuals with the clinical description of 21 Amish patients by Proukakis et al^5^ (Table 1). All affected individuals in the Omani cohort presented with short stature and dysarthria, and were delayed in reaching motor and cognitive developmental milestones. They all had some difficulties walking, with clumsy, mildly spastic gait, which their parents reported to be worsening over time. The most common physical features were relative hypertelorism and overgrowth of the maxilla leading to overbite, as well as hand and feet anomalies such as brachydactyly (5/6 patients), hammer toes, and pes cavus (Fig 2). Additional nonspecific skeletal malformations observed in the hands were clinodactyly, camptodactyly, and hypoplastic 5th middle phalanges (see Fig 2E, F). Most affected individuals had persistent cognitive deficits and poor performance in school, but no emotional lability was reported. Detailed neuropsychological testing was not available.

**Table 1:** Clinical Features of the Omani Troyer Syndrome Individuals and Comparison with the Amish Cohort

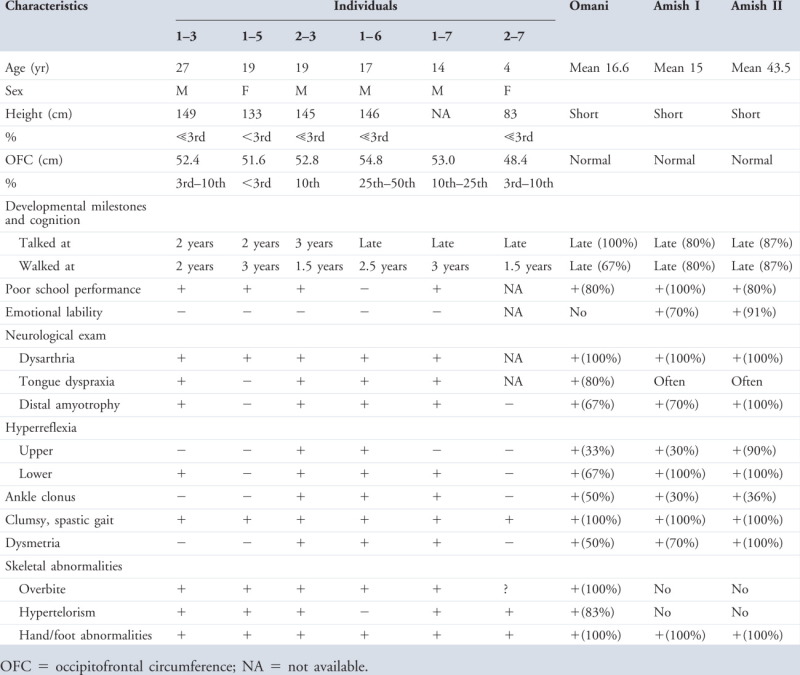

The neurological examination revealed distal amyotrophy (4/6 patients), dysmetria in the upper extremities (3/6 patients), hyperreflexia, which was more severe in the lower limbs (4/6 patients), and ankle clonus (3/6 patients). Heel cords were tight in the 4 older affected individuals. Brain magnetic resonance imaging (MRI) was performed in 2 individuals (1-7 at 14 years and 2-3 at 19 years). Both individuals had mild atrophy of the cerebellar vermis, mild white matter volume loss, and revealed periventricular white matter hyperintensity in T2-weighted images, consistent with gliosis (see Fig 2I, J).

The mean age of our cohort (16.6 years) was younger than the Amish cohort, which might explain the milder and more variable phenotype observed in the Omani individuals; therefore, we subdivided the Amish cohort into a younger subset (Amish I) comparable in age to our cohort (<27 years; mean age, 15 years) and an older subset (Amish II) (>28 years; mean age, 43.5 years). Our cohort closely matched the description of the Amish I group, whereas the Amish II group was more severely affected, consistent with the progressive, degenerative aspects of Troyer syndrome (see Table 1).

### Expression of Spg20 in the Developing and Adult Nervous System

**FIGURE 3:**
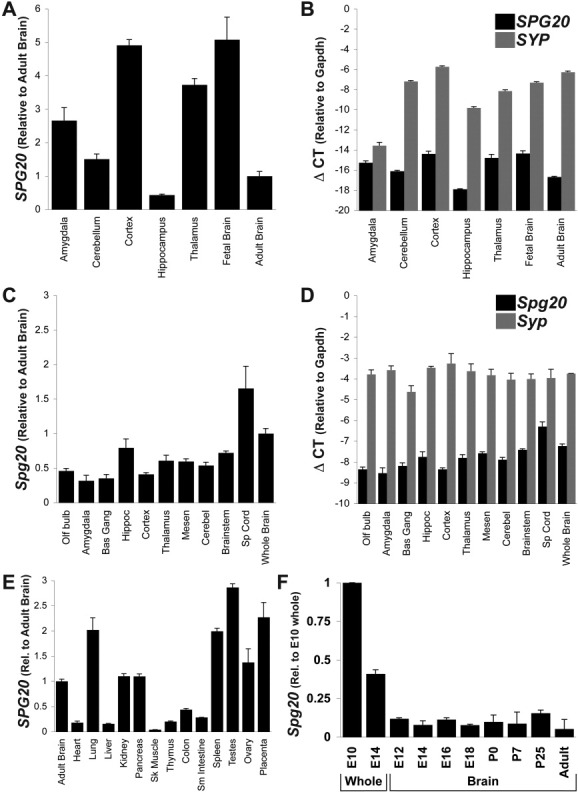
Quantitative polymerase chain reaction analysis of *SPG20*/*Spg20* expression in human and mouse tissues. (A) There is modest variation in *SPG20* local expression in the adult human brain when distinct regions are compared to whole brain samples. (B) Human *SPG20* expression is substantially lower than that of the brain-specific gene synaptophysin (*SYP*) across all brain regions with the exception of the amygdala. (C) A similar profile of regionally variable *Spg20* expression, with some modest differences, is seen in the mouse brain. (D) Lower *Spg20* expression is observed when compared with *Syp*. (E) *Spg20* is expressed in several murine tissues beside the brain. (F) *Spg20* is developmentally regulated in the mouse embryo with highest expression levels in the whole embryo at E10.5. ΔCT =; Bas Gang = basal ganglia; DeltaCT = signal magnitude expressed by the difference in CT values. Hippoc = hippocampus; Mesen = mesenchyme; Cerebel = cerebellum; Sp Cord = spinal cord; Sk Muscle = skeletal muscle; Rel. = relative.

There appear to be 2 major components in Troyer syndrome: an early developmental aspect and a neurodegenerative process. The combination of phenotypes, including limb, craniofacial, and behavioral anomalies, is common in complex genetic disorders affecting early morphogenetic events,^18^ and the relevant genes are often specifically expressed in the embryonic rudiments of the affected adult structures. We first analyzed *SPG20* expression in the developing and adult human brain using qPCR. *SPG20* was expressed at modest levels in the fetal and adult human brain compared to a highly expressed neurally specific gene, synaptophysin (*SYP*; Fig 3A, B). Levels of *SPG20* were highest in the amygdala, cortex, and thalamus, and lowest in the hippocampus and cerebellum. Although clearly detectable, *SPG20* was expressed at substantially lower levels than *SYP*. Expression levels and distribution of the mouse orthologue of *SPG20*, *Spg20,* in the adult mouse brain parallel those of the human, with some divergence; particularly, relative expression of *Spg20* in the hippocampus was slightly elevated compared with humans. In addition, we analyzed *Spg20* expression in mouse spinal cord and brainstem and found that it was highest in the spinal cord of all brain regions measured. As in the human, *Spg20* was expressed at comparatively lower levels than *Syp*. *Spg20* is not a brain-specific gene, as it is also expressed in several other organs.^10^, ^19^ It is, however, developmentally regulated, as expression was maximal at midgestation and embryonic day (E)10, and declined precipitously thereafter (see Fig 3C–F).

**FIGURE 4:**
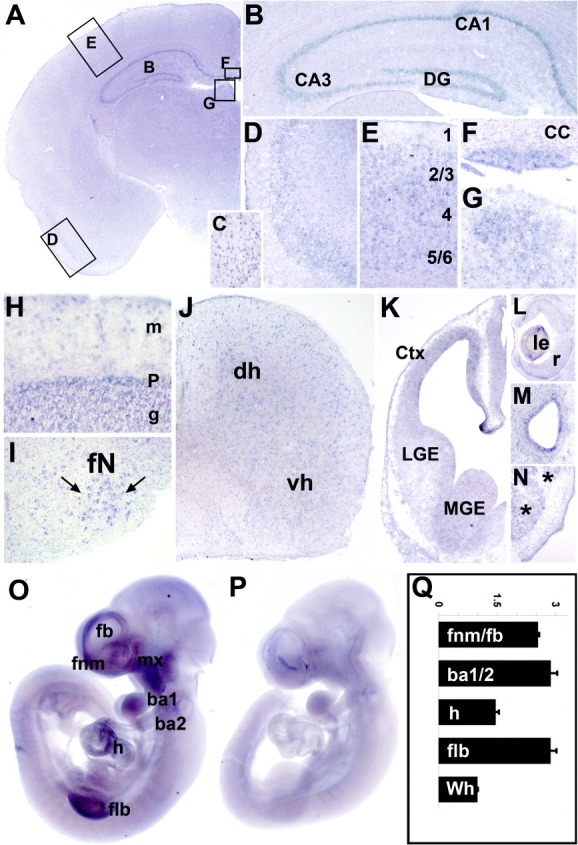
In situ analysis of *Spg20* expression in mice. (A–J) *Spg20* expression in the adult brain is modest and focal. (E) Cortical expression is moderate and not layer-specific. Relatively higher expression is observed in (B) the hippocampus, (C) glial cells in the corpus callosum, (D) entorhinal cortex, (F, G) habenular complex, (H) cerebellum, (I) facial nucleus (fN), and (J) spinal cord. (K) Fetal expression is also modest and distributed in the brain, with higher expression in (L) the lens placode (le), (M) the cochlear epithelium, and (N) condensing mesenchyme at sites of myogenic and cartilage formation (*asterisks*). (O) Early embryonic expression (embryonic day 10.5) is highly patterned and enhanced in the forebrain (fb), frontonasal mass (fnm), maxilla (mx), branchial arches (ba1, ba2), heart (h), and limb buds (flb). (P) The antisense control is completely unlabeled. (Q) Selective, focally elevated expression in the fnm/fb, flb, and ba1/ba2 is confirmed by qPCR after microdissection compared to the heart (h) and whole embryo (Wh). CA1 =; CA2 =; CA3 =; CC = corpus callosum; m = molecular layer; P = Purkinje cells layer; g = granule cell layer; dh = dorsal horn; vh = ventral horn; Ctx = cortex; LGE = lateral ganglionic eminence; MGE = medial ganglionic eminence; r = retina.

We then used in situ hybridization to localize *Spg20* in adult, fetal, and embryonic mouse brain (Fig 4). *Spg20* was expressed at relatively low levels in neurons and glia throughout the adult brain, including glia in fiber tracts. Modestly elevated expression was seen throughout hippocampal stratum pyramidale of the CA fields and dentate gyrus and the transitional parahippocampal/entorhinal cortex. Low expression was seen in the cerebral cortex with no apparent laminar or cell class specificity. The only additional site of elevated expression in the forebrain was the habenular complex, including the habenular recess appended to the corpus callosum. *Spg20* is expressed throughout the cerebellum in Purkinje cells, granule cells, and scattered cells in the molecular layer. In the brainstem, there is robust expression in large neurons, probably motor neurons, and in the facial nucleus. *Spg20* is also expressed in cells distributed throughout the spinal cord with no noticeable discontinuities. As predicted from the qPCR data, expression in the fetal brain is relatively low, with no apparent regional distinctions. Aside from elevated expression in the lens placode and pigment epithelium, cochlear epithelium, and condensing mesenchyme at sites of myogenic or cartilage formation, there is little focal expression at fetal stages (see Fig 4L–N; E12.5 and E14.5 are shown).

In contrast to the later stages, and, as predicted from the qPCR analysis, *Spg20* expression in the midgestation embryo is elevated and highly patterned. In the E10.5 embryos, shortly after neural tube closure, *Spg20* is specifically expressed in the initial frontonasal mass/forebrain, craniofacial structures, aortic arch/heart primordium, and limb buds during morphogenesis, with lowest expression in the heart (Fig 4O, P). qPCR analysis confirms this distribution; expression levels in limb buds, branchial arches, and frontonasal mass/forebrain are substantially elevated compared with the heart as well as with whole E10.5 embryo (Fig 4R). Our observations suggest that *Spg20* has its most specific and maximal activity in the limbs, face and forebrain during early morphogenesis. This is remarkable, because the sites of phenotypic manifestations in the affected members of the Omani Troyer syndrome kindred described here include anomalies of the limb extremities, face, and brain.

## Discussion

Our identification of a novel, disease-associated *SPG20* mutation in an Omani kindred indicates for the first time that Troyer syndrome is not restricted to the Amish, as previously proposed.^2^ Complicated autosomal recessive HSPs are heterogeneous disorders often lacking clear clinical and molecular diagnostic guidelines. Of the 17 distinct loci for complicated HSP identified to date, more than half have been described in single families or isolated populations.^1^, ^2^ In the past 4 decades, several HSP syndromes with Troyer-like features have been reported, but none matched the clinical presentation in the Amish cases.^5^ Some of these disorders were later mapped to different loci such as *SPG26*,^6^, ^20^ *SPG39*,^21^ and *ARSACS*.^22^, ^23^ Even within the isolated region where these Omani families reside, we had described a similar extended pedigree with dysarthria, mental retardation, cerebral palsy, and microcephaly.^24^ Linkage analysis ruled out the *SPG20* locus in the second kindred, confirming that the 2 disorders are separate clinical and genetic entities (A.R., R.S.H., C.A.W., unpublished data). Four individuals in this extended family (N = 45 individuals) also were heterozygous carriers for the *SPG20* mutation, highlighting its presence in this isolated population. Thus, this novel mutation in *SPG20* is geographically and genetically distinct from that in the Amish population; nevertheless, both mutations result in the phenotypic spectrum associated with Troyer syndrome.

Although a clear Troyer syndrome diagnosis is difficult to reach in young subjects due to the variability and mildness of their symptoms, direct comparison of clinical features showed that the Omani cohort closely resembled the age-matched Amish Troyer syndrome group (Amish I), suggesting that *SPG20* null mutations cause a well-defined phenotype. As in the Amish, we observed short stature, skeletal abnormalities in the extremities, developmental delay, and dysarthria of possible cerebellar origin from an early age. Therefore, a diagnosis of Troyer syndrome must be considered when this constellation of phenotypes is present in young children. Facial dysmorphism and skeletal features are subtle, and in single cases in nonconsanguineous populations this disorder could be initially diagnosed simply as cerebral palsy. Spasticity and distal amyotrophy appeared in the teenage years and worsened slowly over time. In the Amish, brain MRI revealed white matter abnormalities, which were less severe in the youngest patient examined (age 15 years), suggesting a progressive worsening of the condition.^5^ MRI analysis indicates that the Omani Troyer syndrome individuals of comparable ages also have mild white matter abnormalities, supporting the hypothesis that white matter degeneration accompanies disease progression, although direct neuronal degeneration cannot be ruled out. In addition, we identified mild cerebellar atrophy, which is consistent with the cerebellar signs. Serial MRI scans of the Omani individuals might resolve whether progressive neurological symptoms are correlated with increased white or gray matter degeneration. Despite many similarities to the Amish cohort, a few specific differences were observed in the affected Omani individuals, including the presence of hypertelorism and a pronounced overbite, and the absence of inappropriate emotional responses. As new cases are identified it will be interesting to assess whether these traits have variable penetrance or are population specific.

Functional studies on spartin, the protein encoded by *SPG20,* have identified multiple roles in protein ubiquitination^25^–^27^ and lipid droplet formation,^19^, ^25^, ^26^ and a possible link to endothelial growth factor receptor signaling.^27^ Previous expression analyses by Northern blot or qPCR identified widespread *SPG20*/spartin localization in the brain and in other tissues^10^, ^19^; however, it was unclear whether regional and cellular dynamism in *SPG20* expression could explain the phenotypes observed in the affected individuals. Our study shows that whereas *SPG20*/*Spg20* expression is modest and virtually ubiquitous in the adult and developing brain, early stages of embryonic development show maximal levels of *Spg20* in the limbs, face, and forebrain primordia. This localized expression suggests a parallel role for *SPG20*/*Spg20* in morphogenesis and differentiation at these phenotypic sites; accordingly, a loss of function mutation may contribute to the phenotypic spectrum of Troyer syndrome.

## References

[1] Harding AE (1983). Classification of the hereditary ataxias and paraplegias. Lancet.

[2] Depienne C, Stevanin G, Brice A, Durr A (2007). Hereditary spastic paraplegias: an update. Curr Opin Neurol.

[3] Salinas S, Proukakis C, Crosby A, Warner TT (2008). Hereditary spastic paraplegia: clinical features and pathogenetic mechanisms. Lancet Neurol.

[4] Cross HE, McKusick VA (1967). The Troyer syndrome. A recessive form of spastic paraplegia with distal muscle wasting. Arch Neurol.

[5] Proukakis C, Cross H, Patel H (2004). Troyer syndrome revisited. A clinical and radiological study of a complicated hereditary spastic paraplegia. J Neurol.

[6] Farag TI, el-Badramany MH, al-Sharkawy S (1994). Troyer syndrome: report of the first “non-Amish” sibship and review. Am J Med Genet.

[7] Farah S, Sabry MA, al-Shubaili AF (1997). Hereditary spastic paraparesis with distal muscle wasting, microcephaly, mental retardation, arachnodactyly and tremors: new entity?. Clin Neurol Neurosurg.

[8] Auer-Grumbach M, Fazekas F, Radner H (1999). Troyer syndrome: a combination of central brain abnormality and motor neuron disease?. J Neurol.

[9] Bertini E, Sabatelli M, Di Capua M (1998). Familial spastic paraplegia, axonal sensory-motor polyneuropathy and bulbar amyotrophy with facial dysmorphia: new cases of Troyer-like syndrome. Eur J Paediatr Neurol.

[10] Patel H, Cross H, Proukakis C (2002). SPG20 is mutated in Troyer syndrome, an hereditary spastic paraplegia. Nat Genet.

[11] Bakowska JC, Wang H, Xin B (2008). Lack of spartin protein in Troyer syndrome: a loss-of-function disease mechanism?. Arch Neurol.

[12] Kuczmarski RJ, Ogden CL, Grummer-Strawn LM (2000). CDC growth charts: United States. Adv Data.

[13] Hall J, Allanson J, Gripp K, Slavotinek A (2007). Handbook of physical measurements.

[14] Leykin I, Hao K, Cheng J (2005). Comparative linkage analysis and visualization of high-density oligonucleotide SNP array data. BMC Genet.

[15] Gudbjartsson DF, Jonasson K, Frigge ML, Kong A (2000). Allegro, a new computer program for multipoint linkage analysis. Nat Genet.

[16] Maynard TM, Meechan DW, Dudevoir ML (2008). Mitochondrial localization and function of a subset of 22q11 deletion syndrome candidate genes. Mol Cell Neurosci.

[17] Meechan DW, Maynard TM, Gopalakrishna D (2007). When half is not enough: gene expression and dosage in the 22q11 deletion syndrome. Gene Expr.

[18] LaMantia AS (1999). Forebrain induction, retinoic acid, and vulnerability to schizophrenia: insights from molecular and genetic analysis in developing mice. Biol Psychiatry.

[19] Robay D, Patel H, Simpson MA (2006). Endogenous spartin, mutated in hereditary spastic paraplegia, has a complex subcellular localization suggesting diverse roles in neurons. Exp Cell Res.

[20] Wilkinson PA, Simpson MA, Bastaki L (2005). A new locus for autosomal recessive complicated hereditary spastic paraplegia (SPG26) maps to chromosome 12p11.1-12q14. J Med Genet.

[21] Rainier S, Bui M, Mark E (2008). Neuropathy target esterase gene mutations cause motor neuron disease. Am J Hum Genet.

[22] Bouchard JP, Barbeau A, Bouchard R, Bouchard RW (1978). Autosomal recessive spastic ataxia of Charlevoix-Saguenay. Can J Neurol Sci.

[23] Engert JC, Berube P, Mercier J (2000). ARSACS, a spastic ataxia common in northeastern Quebec, is caused by mutations in a new gene encoding an 11.5-kb ORF. Nat Genet.

[24] Rajab A, Yoo SY, Abdulgalil A (2006). An autosomal recessive form of spastic cerebral palsy (CP) with microcephaly and mental retardation. Am J Med Genet A.

[25] Eastman SW, Yassaee M, Bieniasz PD (2009). A role for ubiquitin ligases and Spartin/SPG20 in lipid droplet turnover. J Cell Biol.

[26] Edwards TL, Clowes VE, Tsang HT (2009). Endogenous spartin (SPG20) is recruited to the endosomes and lipid droplets and interacts with the ubiquitin E3 ligases AIP4 and AIP5. Biochem J.

[27] Milewska M, McRedmond J, Byrne PC (2009). Identification of novel spartin-interactors shows spartin is a multifunctional protein. J Neurochem.

