# Supplementary material for: Developmental and Degenerative Features in a Complicated Spastic Paraplegia
Source: Ann Neurol. 2009 Nov 30;67(4):516–25. doi: 10.1002/ana.21923 (PMC3027847; doi:10.1002/ana.21923)
Supplement: Supplementary file 1 [file ana0067-0516-SD1.doc]

# Supplementary Table. qPCR primers

|  | Primer sequence | | Amplicon size | |
| --- | --- | --- | --- | --- |
| Human | Forward | Reverse | cDNA | Genomic |
| *SPG20* | ttgccttggtccaactgtttcacatc | gtcctcccgggtttcttcaggtttg | 337 | 2198 |
| *SYP* | gctacgggcctcagggcgactatg | gcagggaaagggacggggtaagag | 330 | 2484 |
| *GAPDH* | gccagccccagcgtcaaaggtg | ctccgggaaactgtggcgtgatg | 328 | 328 |
| Mouse |  |  |  |  |
| *Spg20* | gcaaccggaggagcagccaaagtc | agcttctcctgcgttgtgcccatac | 312 | 7782 |
| *Syp* | tgcgcccacctccttctccaatc | ggacagggctggggaaccgatag | 356 | 3258 |
| *Gapdh* | ctgacgtgccgcctggagaaa | gttgggggccgagttgggatagg | 348 | 348 |
